# A Novel Calcium-Activated Potassium Channel Controls Membrane Potential and Intracellular pH in *Trypanosoma cruzi*

**DOI:** 10.3389/fcimb.2019.00464

**Published:** 2020-01-15

**Authors:** Patricia Barrera, Christopher Skorka, Michael Boktor, Noopur Dave, Veronica Jimenez

**Affiliations:** ^1^Departmento de Biología, Facultad de Ciencias Exactas y Naturales, Instituto de Histologia y Embriologia IHEM-CONICET, Facultad de Medicina, Universidad Nacional de Cuyo, Mendoza, Argentina; ^2^Department of Biological Science, College of Natural Sciences and Mathematics, California State University Fullerton, Fullerton, CA, United States

**Keywords:** potassium channel, intracellular calcium, membrane potential, electrophysiology, *Trypanosoma cruzi*

## Abstract

*Trypanosoma cruzi* develops in environments where nutrient availability, osmolarity, ionic concentrations, and pH undergo significant changes. The ability to adapt and respond to such conditions determines the survival and successful transmission of *T. cruzi*. Ion channels play fundamental roles in controlling physiological parameters that ensure cell homeostasis by rapidly triggering compensatory mechanisms. Combining molecular, cellular and electrophysiological approaches we have identified and characterized the expression and function of a novel calcium-activated potassium channel (TcCAKC). This channel resides in the plasma membrane of all 3 life stages of *T. cruzi* and shares structural features with other potassium channels. We expressed TcCAKC in *Xenopus laevis* oocytes and established its biophysical properties by two-electrode voltage clamp. Oocytes expressing TcCAKC showed a significant increase in inward currents after addition of calcium ionophore ionomycin or thapsigargin. These responses were abolished by EGTA suggesting that TcCAKC activation is dependent of extracellular calcium. This activation causes an increase in current and a negative shift in reversal potential that is blocked by barium. As predicted, a single point mutation in the selectivity filter (Y313A) completely abolished the activity of the channels, confirming its potassium selective nature. We have generated knockout parasites deleting one or both alleles of TcCAKC. These parasite strains showed impaired growth, decreased production of trypomastigotes and slower intracellular replication, pointing to an important role of TcCAKC in regulating infectivity. To understand the cellular mechanisms underlying these phenotypic defects, we used fluorescent probes to evaluate intracellular membrane potential, pH, and intracellular calcium. Epimastigotes lacking the channel had significantly lower cytosolic calcium, hyperpolarization, changes in intracellular pH, and increased rate of proton extrusion. These results are in agreement with previous reports indicating that, in trypanosomatids, membrane potential and intracellular pH maintenance are linked. Our work shows TcCAKC is a novel potassium channel that contributes to homeostatic regulation of important physiological processes in *T. cruzi* and provides new avenues to explore the potential of ion channels as targets for drug development against protozoan parasites.

## Introduction

Ion homeostasis is central to all life forms. Cellular composition must to be regulated in a dynamic manner in order to preserve a relatively constant intracellular environment, even under the most extreme external changes (Pasantes-Morales, 2016). *Trypanosoma cruzi*, the causing agent of Chagas disease, develops in environments with different physiological conditions, including variations in ionic concentrations, pH, and osmolality (Jimenez, 2014). In the hindgut of the insect vector, epimastigotes are exposed to large changes in ionic gradients, including potassium (K^+^), calcium (Ca^2+^) and protons (Kollien et al., 2001). As metacyclic trypomastigotes invade host cells and become intracellular, they also experience significant changes in ionic gradients. Due to the dynamic nature of the environments *T. cruzi* faces throughout its life cycle, it must be able to successfully respond to these environmental changes to ensure survival and propagation between vector and host (Rassi and Marin-Neto, 2010). Ion channels play a role in controlling a wide array of important physiological processes including membrane potential regulation, pH, cell volume, cell proliferation, and death (Lang et al., 2007; Bae et al., 2011; Pasantes-Morales, 2016). They are also validated targets for treatment of highly prevalent diseases such as cardiovascular pathologies (Gill et al., 1992; Turley et al., 2016) and are currently being re-evaluated as potential drug targets against parasitic infections (Meier et al., 2018). In protozoans, ion channel characterization lags behind the general progress of the field, mostly due to technical limitations for direct electrophysiological recordings in motile cells, but in recent years we have gained insight into the role of calcium channels in trypanosomes (Chiurillo et al., 2017; Huang and Docampo, 2018; Potapenko et al., 2019; Rodriguez-Duran et al., 2019).

K^+^ channels are a diverse group of well-characterized ion channels expressed in many different organisms, from bacteria to eukaryotes (MacKinnon, 2003). One important class of K^+^ channels are the calcium-activated potassium channels (CAKC). CAKCs regulate membrane potential (Gui et al., 2012; Alix et al., 2014; Rohmann et al., 2015; Yang, 2016), cell volume regulation and renal K^+^ excretion (Latorre et al., 2017; Sforna et al., 2018) among other cellular functions.

CAKCs are formed by α-subunits with six to seven transmembrane domains, which tetramerize to create the pore-forming region of the channel (Lee and Cui, 2010). This class of channels can be divided into three subclasses by their sequence homology and biophysical properties (Prole and Marrion, 2012). The large conductance (BK) subclass of channels are characterized by ion conductance around 300 pS, voltage sensitivity and activation by Ca^2^ binding to the RCK “calcium bowl” domain of the protein (Horrigan and Aldrich, 2002; Hite et al., 2017). The second subclass is the small conductance (SK) channels, which are characterized by a conductance between 10 and 25 pS, and activation through calcium-calmodulin binding domains (Bond et al., 1999). The final subclass is the intermediate conductance (IK) channels, which activate like SK channels, but their conductance varies between that of BKs and SKs (Kaczmarek et al., 2017; Sforna et al., 2018). *In silico* analysis of Trypanosoma genomes reveals the presence of putative CAKCs (Prole and Marrion, 2012), but homology analysis failed to identify other type of K^+^ channels or accessory subunits usually required for channel trafficking and function. Steinmann et al. showed the role of a heteromeric potassium channel in *T. brucei* membrane potential maintenance (Steinmann et al., 2015) and the presence of a K^+^ channel with atypical features, found in the acidocalcisomes of *T. brucei* (Steinmann et al., 2017). We have previously characterized a non-selective cation channel and its participation in cell volume regulation in *T. cruzi* (Jimenez and Docampo, 2012). Additionally, membrane vesicles isolated from epimastigotes and reconstituted in liposomes showed the presence of, at least, two K^+^ permeable pathways (Jimenez et al., 2011), but the precise nature of the channels responsible for these currents remained elusive. Here, we describe the identification, molecular characterization and physiological role of a novel calcium-activated potassium channel (TcCAKC) in *T. cruzi*. This channel shares structural and functional features with other CAKCs, regulates key physiological parameters such as membrane potential, and is essential for parasite infectivity.

## Materials and Methods

### Sequence Analysis and Structure Prediction

Putative sequences for CAKC channels were identified in Trytrypdb.org (Aslett et al., 2009). Predicted protein sequences for TcCAKC CL Brener Esmeraldo-like (TcCLB.506529.150) and Non-Esmeraldo-like haplotypes (TcCLB.510885.60) were compared with the putative sequences for *Trypanosoma brucei* (Tb927.1.4450) and *Leishmania major* (LmjF.20.0090) homologs. Multisequence alignments and sequence similarity analysis were performed in Geneious Prime with Clustal Omega BLOSUM62 (www.geneious.com). Topology predictions were done comparing the transmembrane domain predictions of TopPred, TMpred, and TMHMM 2.0 (https://www.expasy.org/tools/). Putative calmodulin binding domains were identified using EML (http://elm.eu.org/search.html) and Calmodulin Target Database (http://calcium.uhnres.utoronto.ca/ctdb/ctdb/sequence.html).

### Localization

A fragment of 387 bp of TcCAKC was amplified with primers 5′ ATGAAGGGGGGAGACAATA 3′ and 5′ TTAGGGGTGTTTCCGCACAA 3′ and cloned into pET28(a) with restriction sites BamHI and NotI. The plasmid was transformed in *E. coli* pLys-S for expression of a His-tagged fragment of TcCAKC. After purification with Ni-Agarose, the recombinant protein was injected in rabbits to obtain polyclonal antibodies against the channel (Cocalico). The affinity purified final bleeds were used for immunofluorescence assays in the parasites. Briefly, epimastigotes, bloodstream trypomastigotes and amastigotes were fixed for 30 min in 4% paraformaldehyde. Fixed cells were attached to poly-L-lysine-treated glass coverslips for 10 min. Samples were permeabilized with 0.3% Triton X-100 for 3 min, washed in 1x PBS three times and incubated in 50 mM NH_4_Cl for 30 min at room temperature. After blocking overnight at 4°C in 3% bovine serum albumin (BSA) solution, the cells were incubated with antibodies against TcCAKC (1:100), FCaBP (1:1,000), Calmodulin (SIGMA) (1:250) or SSP1 (1:100) as indicated. Anti-SSP1 (# NR-50891) was obtained from BEI Resources, NIAID, NIH. The secondary antibodies were conjugated with Alexa-fluor 488 or 594 (1:3,000) (Thermo Fischer Scientific, Inc., Waltham, MA). Coverslips were mounted with Fluoromount-G^®^ (SouthernBiotech, Birmingham, AL) containing DAPI (5 μg/mL). Immunofluorescence samples were imaged in an Olympus^©^ IX83 inverted microscope system and processed with CellSense Olympus software.

### Cloning and Expression of TcCAKC in Yeast

The complete ORF of TcCAKC was amplified with forward primer 5′ CGGGATCCACCAATGGAGGGGGGAGACAATAC 3′ and reverse primer 5′ GGAATTCCTGTTGCTTTTGGCCATCCG 3′ and cloned into pYES2 vector with restriction sites BamHI and EcoRI (underlined). The gene was verified by primer walking sequencing and transfected into *Saccharomyces cerevisiae* PLY232 (wild-type) and PLY246 (trk1Δ trk2Δ and tok1Δ null mutants) strains kindly provided by Dr. Per O. Ljungdahl (Ludwig Institute for Cancer Research, Sweden) (Bertl et al., 2003) Wild type cells were maintained at 30°C in standard YPD medium and the mutants were supplemented with 50 mM KCl pH 5.8. Transformed cells were selected in synthetic minimal defined medium without uracil, pH 5.8 [SC ura(-) medium] supplemented with 100 mM KCl to maintain the mutant under viable conditions. Positive clones were confirmed by PCR and expression of TcCAKC was induced switching the carbon source from 2% rafinose to 2% galactose. Complementation studies were done seeding serial dilutions of the complemented mutants in SC ura(-)-galactose agar plates without KCl added, keeping them at 30°C for 3–5 days. Wild-type strains transformed with TcCAKC or with the empty vector were used as a control.

### Site Directed Mutagenesis and Expression in Oocytes

Site directed mutagenesis of the selectivity filter was done using GeneArt Site Directed Mutagenesis kit (Invitrogen) following the manufacturer protocol. Primers were designed to replace residue 313 (Y) for alanine (Y313A) (forward primer 5′ ACGATTTCAACGGTTGGCGCGGGAGATATTATTCC 3′ and reverse primer 5′ CACCACTGCTAAAGTTGCCAACCGCGCCCTCTATA 3′) using the ORF of TcCAKC cloned into TOPO-Blunt II as template and mutations were verified by sequencing. For expression in *Xenopus laevis* oocytes, TOPO-Blunt II vector containing the ORFs for TcCAKC or TcCAKC Y313A was purified and linearized with AseI. Coding RNA (cRNA) was obtained by *in vitro* transcription with mMessenger mMachine T7 kit following the manufacturer's protocol (Ambion). The cRNA length and polyadenylation was verified by non-denaturing gel analysis. Injection of 20 ng (20–40 nL) of the cRNA into chemically defolliculated oocytes (EcoCyte Bioscience) was done using the Nano-Inject II system as previously reported (Jimenez and Docampo, 2015). Oocytes were maintained in Barth's solution (88 mM NaCl, 1 mM KCl, 0.33 mM Ca(NO_3_)_2_, 0.41 mM CaCl_2_, 0.82 mM MgSO_4_, 2.4 mM NaHCO_3_, 5 mM HEPES, 0.1 mg/mL penicillin/streptomycin) at 18°C with daily changes of the solution. Recordings were done at 72 h post injection. Oocytes injected with RNase free DEPC water were used as controls.

### Electrophysiological Recordings

TcCAKC activity was evaluated by two-electrode voltage clamp on oocytes expressing TcCAKC or TcCAKC-Y313A using an Oocyte clamp system OC725 (Warner Instruments). Acquisition of data was done at 10 kHz, with Digidata 1550, and analyzed in pClamp 10. Intracellular electrodes were pulled to resistance of 1–4 MOhms and filled with 3 M KCl solution. Before recording, oocytes were placed in ND-96 recording solution (in mM: NaCl 96, KCl 2, CaCl_2_ 1.8, MgCl_2_, Na pyruvate 2.5 mM, HEPES 5 mM pH 7.4). The steady-state current of oocytes were recorded in response to voltage steps between −80 and 40 mV, with a holding potential of −60 mV. All recordings were performed in ND-96 recording solution or ND-96 calcium-free solution containing 1 μM calcium-activated chloride channel blocker 4,4′-Diisothiocyano-2,2′-stilbenedisulfonic acid (DIDS) to reduce background current during recording. Treatment with 1 μM calcium ionophore ionomycin was applied to bath solution during recording to induce activation of K^+^ currents. Pre-incubation of oocytes with 1 μM thapsigargin, a smooth endoplasmic reticulum calcium pump blocker for 30 min was done to test the effects of increased cytosolic free calcium released from intracellular stores. The average current for any voltage pulse was measured using pClamp10 and plotted against the applied voltage pulse. A Students' *t*-test was run between the experimental condition and the control oocytes to compare the average current at each voltage pulse. Differences in reversal potential toward theoretical equilibriums of particular ions can be indicative of the ions permeating across the membrane. To measure the effect of TcCAKC expression on membrane permeability, injected oocytes were held under voltage ramps from −80 to 40 mV and reversal potential differences were calculated by subtracting pre-ionomycin treatment reversal potential from post-ionomycin reversal potential (V_rev_ = V_I_ –V_F_). Statistics were performed by running a one factor ANOVA with a *post-hoc* Bonferroni correction to compare experimental traces of TcCAKC, and TcCAKC Y313A with control injected oocytes.

### Generation of TcCAKC Knockouts and Phenotypic Analysis

#### Homologous Recombination

TcCAKC knockouts were obtained by sequential allelic replacement by homologous recombination. Recombination cassettes were obtained by PCR of neomycin or hygromycin resistance genes flanked by 500 bp of TcCAKC 5′ and 3′ UTRs. The fragments were amplified with allele specific primers and cloned into TOPO-Blunt II vector. Constructs verified by sequencing where purified, linearized, and transfected into CL strain epimastigotes using AMAXA nucleofector system protocol U-033. Selection was carried out with 250 μg/ml of G418 and 100 μg/ml of hygromycin. Once selection was complete, parasite populations were subcloned by serial dilution and screened by PCR to verify the correct insertion of the replacement cassettes using primers annealing upstream and downstream of TcCAKC UTRs.

Level of expression of TcCAKC in single-allele replacement (sKO) and double-allele replacement parasites (dKO) was evaluated by qPCR. Epimastigotes were collected during mid-log phase of growth, washed once with 1x PBS pH 7.4 and homogenized in TRI Reagent^®^. Total mRNA was extracted following the manufacturer protocol (Sigma-Aldrich, St. Louis, MO) followed by chloroform/ ethanol precipitation cDNA was obtained using SuperScript^®^ III First-Strand Synthesis System (ThermoFisher Scientific, Inc., Waltham, MA) and oligo-dT_(20)_ primers. cDNA was analyzed by qPCR with Power SYBR Green PCR Master Mix (Applied Biosystems) and primers forward 5′ GAACGTGGTCGGGTCAATCT 3′ and reverse 5′ GAGGCGACGTGTGTGAGAAT 3′. All qPCR results were normalized against GAPDH and tubulin as housekeeping gene and indicated as ΔΔCq Mean ± SD of at least 3 independent experiments in triplicate.

#### Phenotypic Analysis of Mutants

CL strain epimastigotes were cultured in LIT media supplemented with 10% inactivated Fetal bovine serum (FBS) at 28°C. Knockout parasites were maintained with 250 μg/mL G418 (sKO) plus 100 μg/mL hygromycin (dKO) (Bone and Steinert, 1956). To evaluate the growth of the parasites, cells were diluted to a concentration of 1 × 10^6^/mL in LIT media supplemented with FBS and antibiotics and counted every 24 h for 5 days in a Z2 Cell Counter (Beckman Instruments). Cell counts were taken in triplicate from three independent experiments. Statistics were ran using one factor ANOVA with *post-hoc* Bonferroni test to compare mutant and wild-type (WT) cells. To evaluate the infective capacity of the mutants, differentiation to metacyclic trypomastigote forms was induced under chemically defined conditions using triatomine artificial urine (TAU) medium as described (Contreras et al., 1985). Epimastigotes at 4 days of growth were collected by centrifugation at 1,600 × g for 10 min, washed once in phosphate buffered saline solution (PBS) pH 7.4, resuspended in TAU media and incubated 2 h at 28°C. The supernatant was collected and resuspended in TAU with amino acids (TAU3AAG), incubated for up to 7 days at 28°C, collected by centrifugation resuspended in 5 mL of Dulbecco's Modified Eagles Media (DMEM) supplemented with 20% fresh FBS to eliminate residual epimastigotes.

#### In vitro Infection Assays

HEK-293 cells were plated onto coverlips in 12 well plates (1,000 cells/well) and incubated in supplemented HG-DMEM overnight at 37°C with 5% CO_2_. Infections were performed at a multiplicity of infection (MOI) of 25:1 with either WT, sKO or dKO mutant trypomastigotes. After 6 h, the cells were washed 3 times with Hank's media and fresh DMEM was added. Coverslips were fixed in 4% paraformaldehyde-PBS at 6, 24, and 48 h, stained with DAPI (5 μg/ml) and mounted in Fluoromont media for quantification of intracellular parasites. All infection quantifications were done in 4 coverslips per experiment, in 3 or more independent experiments. At least 100 host cells were quantified per coverslip. The number of host cells vs. parasites was compared by Student *t*-test.

### Fluorometric Measurements

All fluorometric measurements were done in WT, sKO, and dKO TcCAKC epimastigotes collected at 4 days of growth. Cells were pelleted at 1,600 g for 10 min at room temperature, washed three times with Buffer-A with Glucose (BAG: in mM NaCl 116, KCl 5.4, MgSO_4_ 0.8, glucose 5, HEPES 50 pH 7.3) and resuspended at a density of 1 × 10^9^ cells/mL in the appropriate buffer for the measurement. Recordings were done on a Hitachi F7000 spectrofluorometer. For all the experiments, ionic replacement was done by substituting Na^+^, K^+^ or both by N-Methyl-D-glucamine (NMDG) in the corresponding standard buffer.

#### Membrane Potential

Aliquots of 1 × 10^8^ cells were diluted in Standard buffer (in mM: NaCl 135, KCl 5, CaCl_2_ 1, MgSO_4_ 1, glucose 5, HEPES 10 pH 7.4) plus 1 μM DisBac_2_(3). Fluorescence was recorded at 1 Hz with excitation at 530 nm and emission at 560 nm. Calibration was performed by adding 1 μM of gramicidin to WT epimastigotes in NMDG Buffer (composition) and increasing concentrations of K^+^ gluconate (0.1, 1, 2, 5, 10, 25, 50, 100 mM). Calibration potentials were calculated using the theoretical Nernst potential equation $\left[V_{eq}=\frac{RT}{zF}\ln(\frac{{\left[K^{+}\right]}_{out}}{{\left[K^{+}\right]}_{in}})\right]$, where [K^+^]_in_ was assumed to be 120 mM (Van Der Heyden and Docampo, 2002). A linear line of best fit was generated and the equation for this line was used for interpolation of experimental data. Experimental measurements were done as described above. Resting membrane potential was averaged over the first 100 s of baseline recording. All recordings were done in Standard buffer unless otherwise indicated.

#### Intracellular pH

Epimastigotes were loaded with 6 μM BCECF-AM at 30°C for 30 min in standard buffer, washed twice and resuspended at a concentration of 1 × 10^9^ cells/mL in Standard buffer. Fluorescence was measured with excitation wavelengths of 490/440 nm and emission of 530 nm. Recordings were done in Standard Buffer unless otherwise indicated. Calibration was done in high K^+^ Standard Buffer (135 mM KCl, 1 mM MgSO_4_, 1 mM CaCl_2_, 5 mM glucose and 10 mM HEPES-Tris, pH 7.4), with 1 μM nigericin at various pHs (6, 6.5, 7.0, 7.4, 7.6, 8.0). Once stabilized, the fluorescent reading for any pH was averaged over 100 s and plotted against pH. The linear fit was then used to interpolate the experimental data.

#### Proton Extrusion

Measurements of proton extrusion were done in the presence of 0.38 μM BCECF Free acid mixed with 1 × 10^8^ epimastigotes in low buffer standard solution (in mM: NaCl 135, KCl 5, CaCl_2_ 1, MgSO_4_ 1, glucose 5, HEPES 0.1 pH 7.4) (Benchimol et al., 1998). The recordings were done at excitation wavelengths of 490/440 nm and emission of 530 nm. Calibration was performed as indicated above. Differences in proton extrusion were measured by looking at differences in the slope over the first 50 s of recording (Initial rate of extrusion) or in the last 200 s of recording (Final). All experiments were done in low buffered standard buffer unless otherwise indicated.

#### Intracellular Calcium

Epimastigotes were loaded with 5 μM Fura2-AM (Molecular Probes) in BAG for 30 min at 30°C, washed twice and resuspended in BAG at a concentration of 5 × 10^8^ cells/ml. Aliquots of 5 × 10^7^ cells were taken for each measurement with excitation at 340/380 nm and emission at 525 nm. Recordings were performed in BAG unless otherwise indicated. Calibration was done by permeabilizing cells in BAG + 1 mM EGTA and then adding increasing concentrations of CaCl_2_. The concentration of free calcium available was calculated using MaxChelator software (https://somapp.ucdmc.ucdavis.edu/pharmacology/bers/maxchelator/CaEGTA-TS.htm) and the Kd was calculated according to the manufacturer protocol. Experimental recordings were allowed to stabilize at baseline before addition of 1.8 mM CaCl_2_ and averaged for 100 s after stabilization. For all the experiments, ionic replacement was done by substituting Na^+^, K^+^ or both by N-Methyl-D-glucamine (NMDG) in the corresponding standard buffer.

## Results

### TcCAKC Shares Structural Features With Other Calcium-Activated Potassium Channels

The genome of *T. cruzi* CL strain contains two sequences that share homology with calcium- activated potassium channels. CL Brener Esmeraldo-like (TcCLB.506529.150) and Non-Esmeraldo-like haplotypes (TcCLB.510885.60) are 94% identical at the protein level and show 45% identity with *T. brucei* TbK1 (Tb927.1.4450) (Steinmann et al., 2015) and 37% identity with *L. major* homolog LmjF20.0090 (Figure S1). BlastP analysis of Trypanosoma sequences only shows significant homology with sequences encoding for calcium-activated potassium channels (CAKCs), although the overall identity is below 20%. Based on this, we named the channel TcCAKC and further analysis revealed conserved features found in other CAKCs. The channel has 6 transmembrane domains, a conserved selectivity filter (TVGYG) in the loop between TM5 and TM6 and multiple putative calmodulin binding sites (Figure S2), previously described as the mechanism that mediates the calcium dependency of intermediate conductance channels (Sforna et al., 2018). Unlike CAKCs of large conductance, TcCAKC does not possess calcium binding sites, suggesting that its activation is rather mediated by Ca^2+^-Calmodulin binding, as it has been shown for IK and SK channels.

### Localization in the Parasites

**Figure 1:**
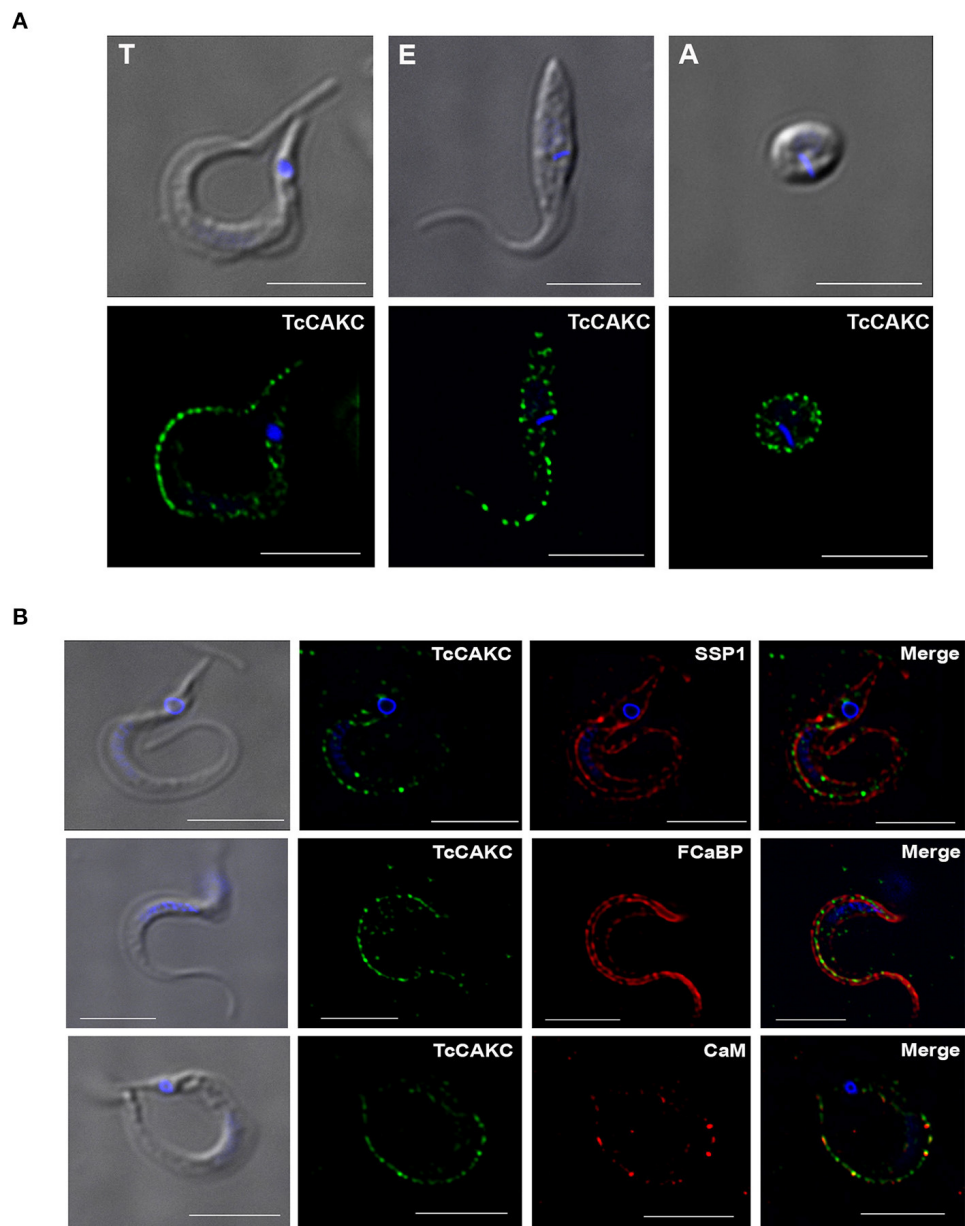
TcCAKC localization. **(A)** Immunofluorescence analysis of *T. cruzi* trypomastigotes (T), epimastigotes (E), and amastigotes (A) with polyclonal antibodies against the channel (green). **(B)** Trypomastigotes immunofluorescence showing TcCAKC (green) colocalization with membrane marker SSP-1 (red), and calcium binding proteins FCaBP and calmodulin. Nuclei and kinetoplasts were DAPI stained. Bar size: 10 μm.

**Figure 2:**
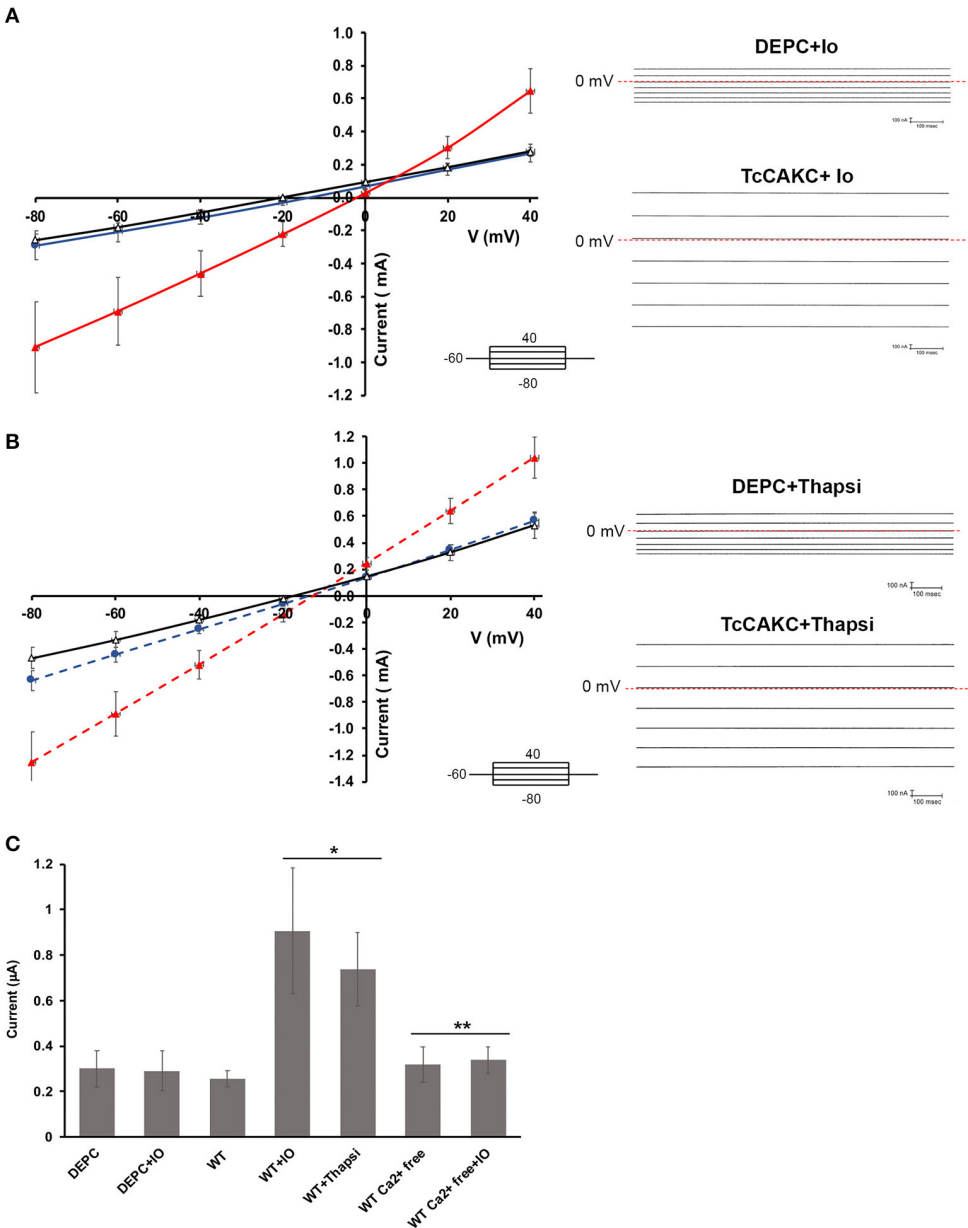
Electrophysiological characterization of TcCAKC**. (A)** Current-voltage relationship of oocytes expressing TcCAKC in the absence (black) or presence (red) of 1 μM ionomycin. Control currents are obtained from oocytes injected with DEPC water and treated with 1 μM ionomycin (blue line). Representative traces are shown in the right panel. Values are Mean ± SD of *n* = 18 oocytes. **(B)** Current-voltage relationship of oocytes expressing TcCAKC (red) or control (blue) preincubated with thapsigargin (*n* = 15). TcCAKC currents in absence of thapsigargin are indicated in black. Representative traces are shown in the right panel. All recordings are in ND-96 buffer with a holding potential of −60 and 20 mV step protocol between −80 and 40 mV. **(C)** Maximum currents at −80 mV (in absolute values) for oocytes under the indicated conditions. Calcium free conditions were achieved by addition of 1 mM EGTA in ND 96 buffer without added CaCl_2_. Values are Mean ± SD of *n* = 15 oocytes. ^*^p < 0.01 respect to the WT, ^**^p < 0.01 respect to the corresponding condition without EGTA.

Immunolocalization analysis with specific antibodies against TcCAKC show a distinct punctate localization in the periphery of trypomastigotes, epimastigotes, and amastigotes (Figure 1A). Co-localization with SSP-1 (Figure 1B), a membrane marker for trypomastigotes, confirms that TcCAKC is expressed at the surface of the parasites. As expected based on the topology and sequence analysis, the channel colocalizes with calmodulin (Figure 2B, bottom panel) and flagellar calcium-binding protein (FCaBP-Figure 2B), an important calcium sensor in the flagellum of trypanosomatids (Buchanan et al., 2005).

### Functional Studies

#### Yeast Complementation

To demonstrate the function of TcCAKC as a potassium channel we expressed the protein in *S. cerevisiae* PLY246 (trk1Δ trk2Δ and tok1Δ null mutant). In this strain, the principal K^+^ permeation pathways have been eliminated and the cells require the supplementation of the media with high amounts of this ion to sustain their growth (Bertl et al., 2003). After 2 h of induction TcCAKC was expressed in the yeast vacuole and at later points (24 h) in the plasma membrane (Figure S3A). Importantly, the channel expression was able to revert the growth phenotype of this mutant providing evidence that it is, in fact, a K^+^ permeable channel (Figure S3B).

#### Electrophysiological Characterization

**Figure 3:**
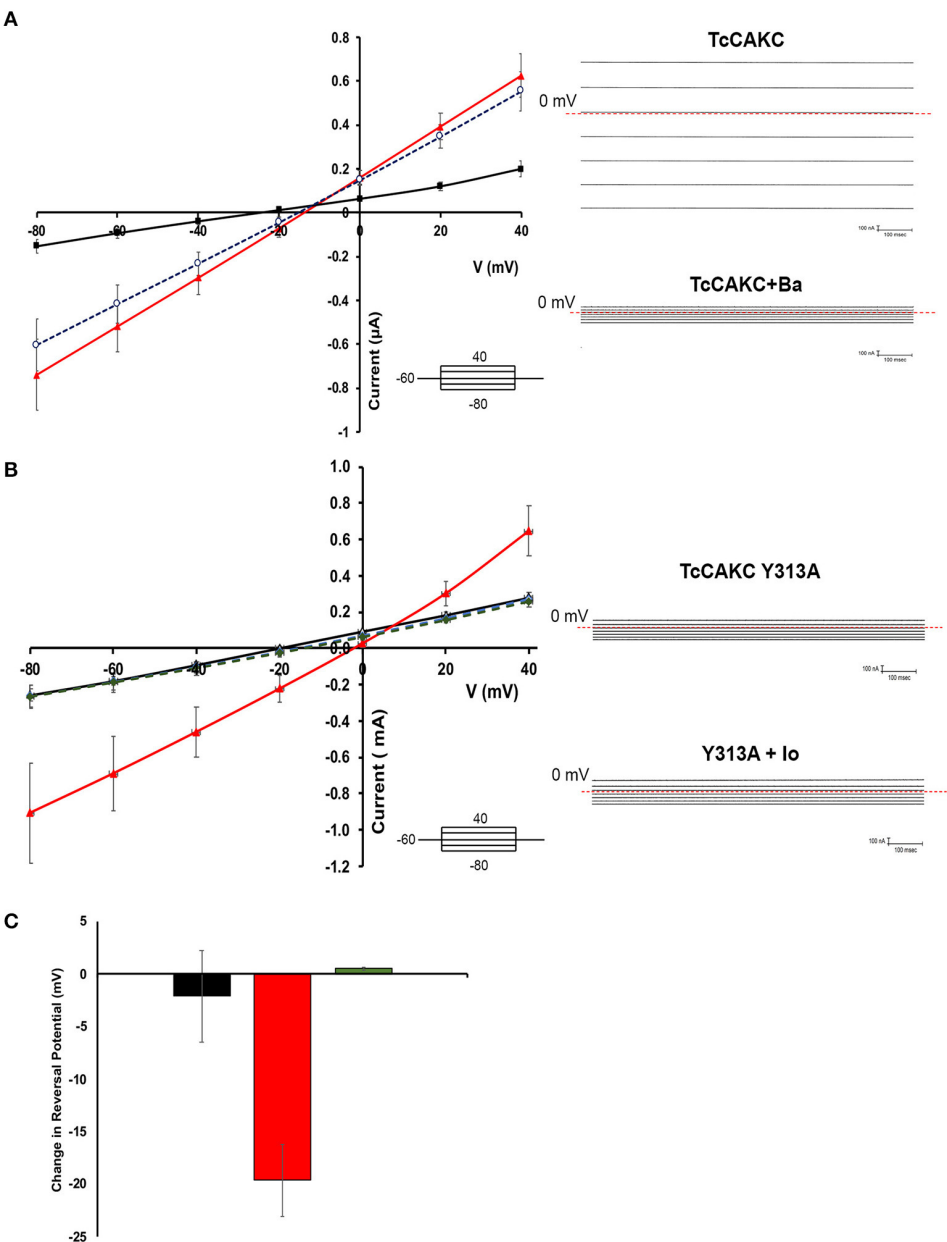
Blockage characteristics of TcCAKC. **(A)** Current-voltage relationship showing the effect of potassium channel blockers on thapsigargin-elicited TcCAKC currents (red line). One millimolar of extracellular BaCl_2_ significantly reduced the current (black line) while up to 300 μM 4-AP had no significant effect. Values are Mean ± SD of *n* = 10 oocytes from 3 independent days or recording. Representative traces are shown in the right panel. **(B)** Current-voltage relationship of oocytes expressing WT TcCAKC in absence (black line) or presence (red line) of ionomycin or TcCAKC-Y313A mutant (doted green and blue lines). Values are Mean ± SD of *n* = 15 oocytes from 3 independent experiments. Representative traces are shown in the right panel. **(C)** Quantification of the reversal potential calculated from the currents obtained under ramp protocols in control oocytes (black), cells expressing TcCAKC (red) or TcCAKC-Y313A (green)Values are Mean ± SD of *n* = 35 oocytes from 7 independent experiments.

Two-electrode voltage clamp recodings of *X. laevis* oocytes expressing TcCAKC showed a stable resting membrane potential of −22.8 ± 7 mV (*n* = 24), similar to the membrane potential of control oocytes injected with DEPC water (−26.8 ± 6 mV, *n* = 24), indicating that the expression of the channel did not significantly affect the health of the oocytes. When cells where subjected to a voltage step protocol from −80 to 40 mV with a holding potential of −60 mV, oocytes expressing TcCAKC (Figure 2A black line) did not show significant differences in their currents compared with control cells (Figure 2A blue line). Addition of ionomycin (IO) did not induce a significant increase in the currents of the control cells, but it elicited a strong current in the TcCAKC expressing cells (Figure 2A red line). Similar activation was observed after incubating the oocytes with thapsigargin to release Ca^2+^ from the endoplasmic reticulum (Figure 2B red line). When extracellular Ca^2+^was chelated by addition of EGTA to the medium, the effect of ionomycin was abolished (Figure 2C), indicating that the activation can be triggered by Ca^2+^ from intracellular stores or by influx from the extracellular media. These results confirm that TcCAKC is able to form functional channels by itself and its activity requires increase in cytosolic Ca^2+^. It is important to point out that all recordings were performed in the presence of 1 μM DIDS to block endogenous currents resulting from the activation of calcium-dependent chloride channels, abundant in *X. laevis* oocytes (Weber W., 1999). TcCAKC activity induced by thapsigargin (Figure 3A red line) was blocked by 1 mM BaCl_2_ (Figure 3A black line) while 4-aminopyridine had no significant effect (Figure 3A blue line). To confirm the selective nature of the channel, we mutated the tyrosine at position 313 for alanine (Y313A) and performed similar experiments as described above. This mutation, located in the middle of the conserved selectivity filter (TVGYG), is predicted to render an inactive channel (Heginbotham et al., 1994; Noskov and Roux, 2006). Indeed, oocytes expressing TcCAKC-Y313A showed no current activation upon treatment with ionomycin (Figure 3B green line).

Reversal potential is the voltage at which there is no net current across the membrane and is indicative of the type of ions permeating through a membrane (Hille, 1978). Voltage ramps between −80 and 40 mV were used to test if TcCAKC activation leads to differences in oocyte permeability to K^+^. TcCAKC expressing oocytes treated with ionomycin had a shift in reversal potential of −19.6 mV (Figure 3C red), while control and TcCAKC-Y313A expressing oocytes had almost no shifts in reversal potential [Figure 3C control −2.14 mV (black) and TcCAKC-Y313A 0.43 mV (green)]. The shift in reversal potential for the TcCAKC oocytes is in the direction of the theoretical Nernst Potential for K^+^ (−80.5 mV, assuming [K^+^]_intra_ of the oocyte is 120 mM, based on previous literature; Weber W. M., 1999), indicates an increase in membrane permeability to K^+^, further confirming TcCAKC as a K^+^ conducting channel.

Overall, the functional complementation of K^+^ deficient yeast and the biophysical characterization of TcCAKC expressed in *X. laevis* oocytes provide solid evidence that this is a calcium-activated potassium channel that can form functional pores without co-expression of additional subunits.

### TcCAKC-KO Impairs Growth and Infectivity in the Parasites

**Figure 4:**
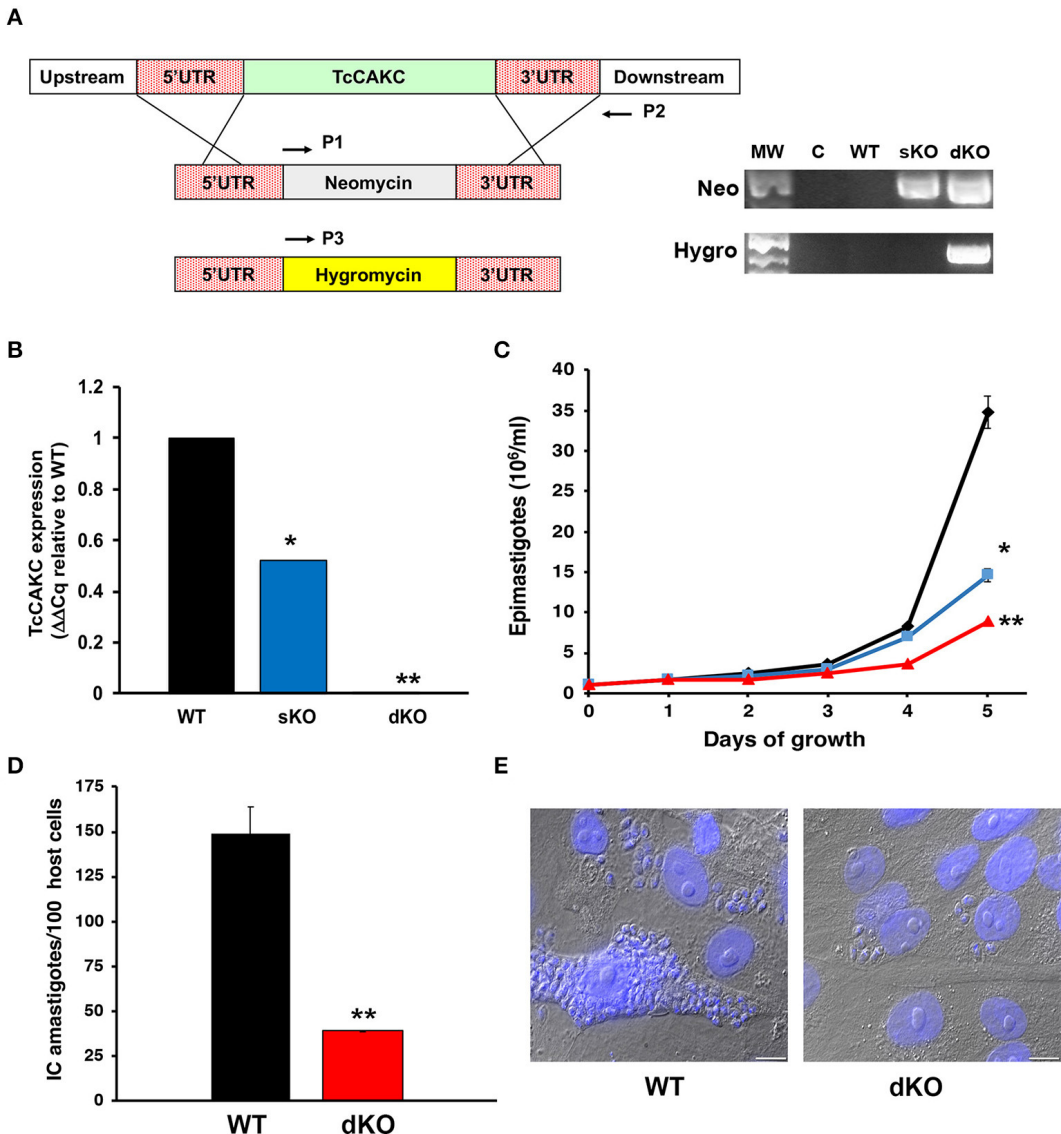
Effect of TcCAKC knockout in *T. cruzi* fitness. **(A)** Schematic representation of the allelic replacement strategy for TcCAKC and genomic DNA screening showing the correct insertion of the drug resistance cassettes, screened with primers P1–P2 (neomycin) and P1–P3 (hygromycin). **(B)** qPCR analysis of TcCAKC expression levels in epimastigotes wild type (WT), one (sKO) or both (dKO) *TcCAKC* alleles replaced. Values are expressed as ΔΔCq and normalized using GAPDH and tubulin as housekeeping genes. Mean ± SD of 3 independent experiments. ^*,**^p < 0.01 respect to WT. **(C)** Growth curve of epimastigotes WT (black), sKO (blue) and dKO (red). Mean ± SD of 3 independent experiments. ^*,**^p < 0.05 respect to WT at day 5 of growth. **(D)** Quantification of intracellular amastigotes comparing WT and dKO parasites at 48 h post-infection. At least 100 host cells were counted in 4 coverslips per experiment, 3 independent experiments. Mean ± SD of 3 independent experiments, ^**^p < 0.01. **(E)** Representative images of infections quantified in **(D)**. The cells were fixed and DAPI stained for quantification.

To evaluate the role of TcCAKC in *T. cruzi*, we sequentially replaced both alleles of the gene by homologous recombination with antibiotic resistance cassettes flanked by 500 bp of the 5′ and 3′UTR of the gene specific for each haplotype (Figure 4A). The introduction of the first replacement cassette (neomycin) eliminated the Esmeraldo-like allele (in chromosome 6s), while the non-Esmeraldo like allele (6p) was replaced by a hygromycin resistance gene (Figure 4A). The level of expression was verified by qPCR (Figure 4B), and as expected, ablation of one allele (sKO) decreased the transcript levels by ~50% while elimination of both (dKO) decreased the mRNA levels more than 95%. As a consequence of *TcCAKC* ablation, the growth of sKO and dKO epimastigotes is severely impaired (Figure 4C) but despite a very low rate of replication, the phenotype is not lethal, suggesting that K^+^ homeostasis is one of many determinants of parasites fitness. Importantly, dKO parasites have a significant decrease in infectivity, with low production of intracellular amastigotes (Figures 4D,E). These infections are non-productive, as the amastigotes fail to differentiate to trypomastigotes and escape the cells. We were not able to recover tissue-derived trypomastigotes from the supernatant of the cultures and all infection assays were done with metacyclic trypomastigotes differentiated *in vitro*. This is not surprising given the fact that intracellular amastigotes develop in a high potassium environment, where TcCAKC seems to be playing an essential role in parasite homeostasis.

### TcCAKC Regulates Key Physiological Parameters

#### Membrane Potential

**Figure 5:**
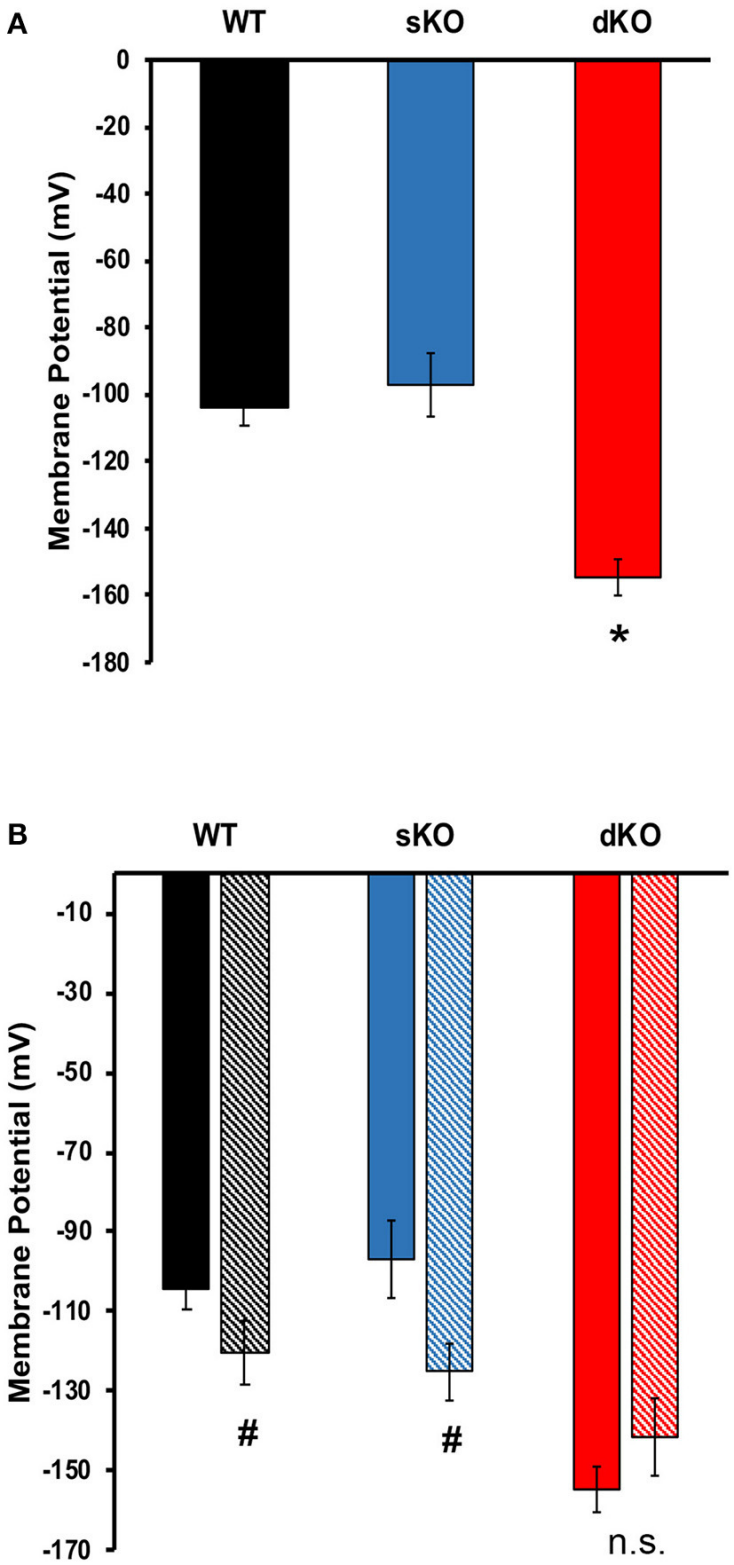
Membrane potential. **(A)** Resting membrane potential of epimastigotes WT (black), TcCAKC sKO (blue) and TcCAKC dKO (red) in standard buffer with 1 μM DisBac_2_(3). **(B)** Resting membrane potential of epimastigotes in standard buffer (solid bars) or NMDG buffer (striped bars). Values represent Mean ± SD of 5 independent experiments. ^*^p < 0.05 compared with WT, ^#^*p* < 0.05 compared with the values obtained in standard buffer.

Since other CAKCs have major influences on membrane potential modulation in eukaryotic cells, it was of interest to elucidate its role in *T. cruzi*. To test this, fluorometric measurements of resting membrane potential and membrane potential responses were performed to compare differences between WT and mutant epimastigotes. In standard buffer, dKO mutants had a hyperpolarized resting membrane potential (−154.7 ± 5.66 mV, *N* = 5) when compared to either the WT parasites (−104.1 ± 5.19 mV, *N* = 5) and sKO parasites (−97.03 ± 9.63 mV, *N* = 5) (Table 1, Figure 5A). This result suggests that TcCAKC plays a role in membrane potential maintenance in *T. cruzi* by allowing the influx of K^+^ to the cells. These results are in agreement with previous research, showing that K^+^ causes depolarization on epimastigotes and suggesting the presence of a K^+^ conducting pathway (Van Der Heyden and Docampo, 2002) responsible for this effect.

**Table 1:** Resting membrane potential.

	**WT (mV)**	**sKO (mV)**	**dKO (mV)**
Standard	−104.05 ± 5.19	−97.03 ± 9.63	−154.70 ± 5.65^*^
Na^+^ free	−101.96 ± 2.70	−102.13 ± 2.67	−154.24 ± 4.88^*^
K^+^ free	−109.20 ± 2.47	−111.30 ± 3.28	−149.65 ± 3.79^*^
NMDG	−120.40 ± 7.95^#^	−125.00 ± 7.21^#^	−141.40 ± 9.81^*^

*Resting membrane potential of epimastigotes in various ionic environments. Epimastigotes were recorded in 1μM DisBac_2_(3). Data is average resting membrane potential from first 100 s of recording. Data presented as M±SD*.

* *p < 0.05 when compared to WT in same conditions*.

# *p < 0.05 when compared to same cell type in standard buffer using a Two-Way ANOVA with post-hoc Bonferroni correction. N = 5 for all conditions*.

The WT and knockout mutants were recorded to ion depleted buffers to test if the extracellular ionic composition has an effect on membrane potential in the parasite. Na^+^ free, and K^+^ free buffers (Table 1) did not have significant effects on the resting membrane potential of any of the cell lines. Replacement of Na^+^ and K^+^ with non-permeating cation N-methyl-D-glucamine (NMDG), produced significant depolarization in WT (−120.40 ± 7.95 mV) and sKO (−125.00 ±7.45 mV) parasites but did not further hyperpolarize the dKO epimastigotes (Figure 5B). These results support previously reported evidence that *T. cruzi* membrane potential is not maintained by Na^+^/K^+^ balance, but is instead primarily dependent on protons (Van Der Heyden and Docampo, 2000). Our results also indicate that, although Na^+^ and K^+^ are not the main driver of membrane potential, the presence of at least one of these ions is required for proper membrane potential homeostasis, presumably to fuel exchangers that contribute to the proton-motive force. To maintain the electrochemical gradients, TcCAKC could be one of the K^+^ influx pathways and its ablation in the dKO parasites pushes the membrane potential to more hyperpolarized values.

#### pH and Proton Extrusion

**Figure 6:**
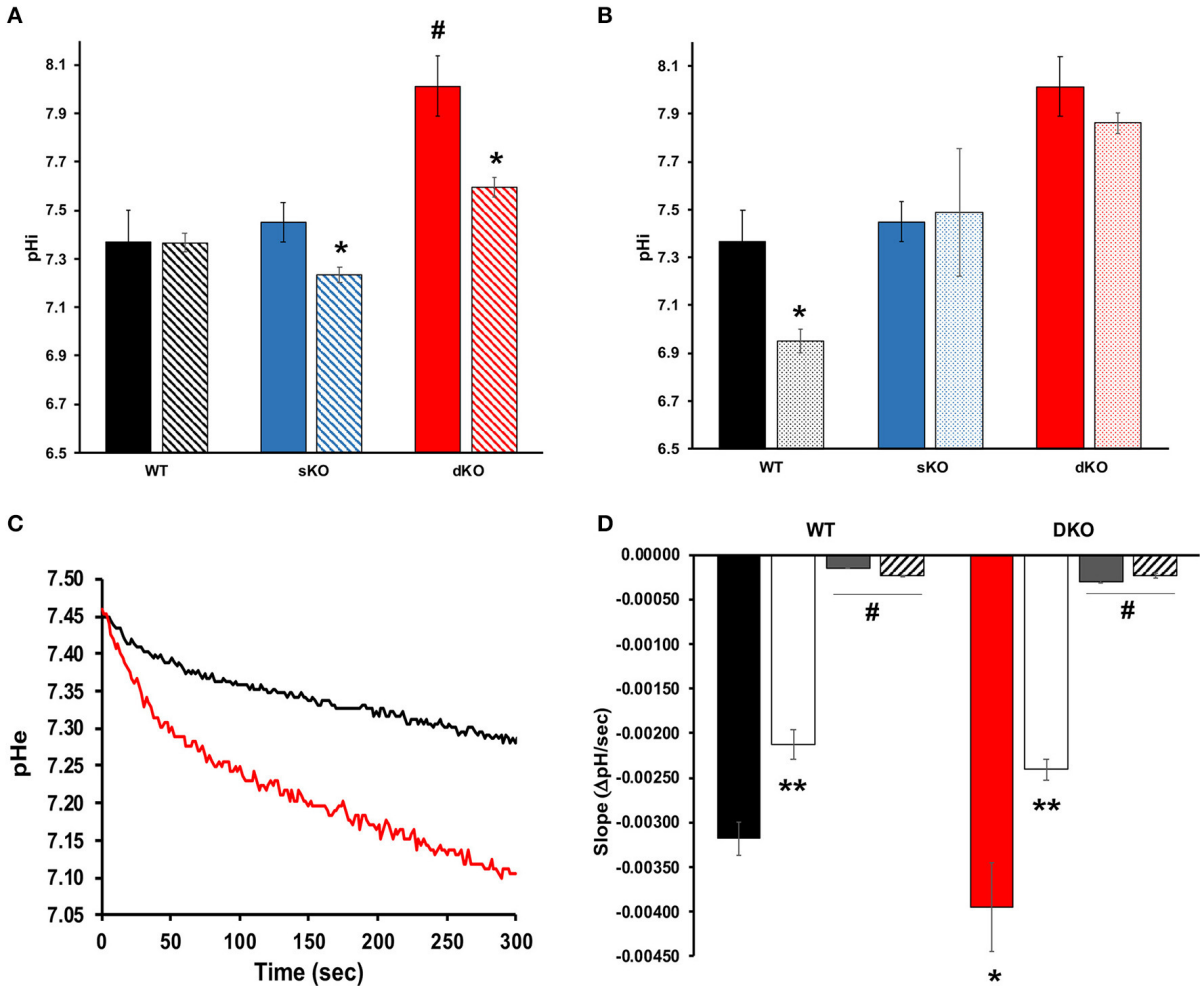
Intracellular pH and proton extrusion. **(A)** Intracellular pH measurements of epimastigotes in standard (solid bars) or NMDG buffer (striped bars). Values represent Mean ± SD of 4 independent experiments. # indicates *p* < 0.05 compared with WT, ^*^p < 0.05 compared with the values obtained in standard buffer. **(B)** Effect of BaCl_2_ on pH regulation. Values of intracellular pH were compared in standard buffer with (dotted bars) or without (solid bars) 1 mM BaCl_2_. ^*^p < 0.05 from 4 independent experiments. **(C)** Representative traces showing proton extrusion in epimastigotes WT (black trace) or TcCAKC dKO (red trace) measured with BCECF free acid. **(D)** Slope analysis for the first 50 s of proton extrusion under standard low buffering solution conditions (WT: black bar, dKO: red bar), K^+^-free (white bars), Na^+^ free (gray bars) or NMDG buffer (striped bars). Values are Mean ± SD of 4 independent experiments. ^*^p < 0.05 compared with WT in standard buffer, ^**^p < 0.05 for each strain in K^+^ free compared with the values obtained in standard buffer, #*p* < 0.05 of Na^+^ free or NMDG buffer compared with standard conditions.

Since TcCAKC KO had an important effect on membrane potential maintenance, and previous studies have described protons as the primary regulator of membrane potential in *T. cruzi* (Van Der Heyden and Docampo, 2000), it was vital to interrogate the role TcCAKC had on intracellular pH (pH_i_) regulation. The pH_i_ of WT (7.36 ± 0.131) and sKO (7.45 ± 0.082) epimastigotes was similar to previously reported values (Van Der Heyden and Docampo, 2000) but dKO parasites had a drastic intracellular alkalinization (8.01 ± 0.124) (Figure 6A solid bars, Table 2). No ionic replacement (Na^+^. K^+^, or NMDG) caused a significant change in the cytosolic pH of the WT parasites, but replacement of both Na^+^ and K^+^ with NMDG caused acidification in sKO and dKO epimastigotes suggesting a less robust homeostatic potential when TcCAKC expression is decreased (Figure 6A, striped bars and Table 2). These results confirm previous findings in *T. cruzi* showing an interdependency of pH and membrane potential, linked via proton regulation (Van Der Heyden and Docampo, 2002; Vieira et al., 2005). As TcCAKC dKO cells present a hyperpolarized membrane potential, this is also transduced in a relative proton deficit and the observed cytosolic alkalinization.

**Table 2:** Intracellular pH of epimastigotes.

	**WT**	**sKO**	**dKO**
Standard	7.36 ± 0.131	7.45 ± 0.082	8.01 ± 0.124^*^
Na^+^ free	7.19 ± 0.241	7.44 ± 0.110	8.02 ± 0.037^*^
K^+^ free	7.58 ± 0.097	7.43 ± 0.044	7.87 ± 0.184
NMDG	7.37 ± 0.072	7.23 ± 0.031^*^	7.59 ± 0.04^*^

*Intracellular pH (pH-_intra_) of epimastigotes in various ionic environments. Measurements made in epimastigotes loaded with BCECF-AM. Data is average pH from first 50 s of recording before any treatment given. Data presented as M±SD*.

* *p < 0.05 when compared to the WT strain in the same condition. Two Factor ANOVA with post-hoc Bonferroni correction. N = 4 for all conditions*.

To further explore the link between TcCAKC-mediated K^+^ influx and pH regulation we performed pH_i_ measurements in standard buffer in the presence of 1 mM BaCl_2_, a blocker that showed a strong effect in the TcCAKC currents in oocytes (Figure 3A). This divalent cation induced a significant acidification in the WT parasites (Figure 6B) but only a marginal effect in the dKO, indicating that the K^+^ influx through the channel is necessary for pH compensatory mechanisms, perhaps by K^+^/H^+^ and Na^+^/H^+^ exchangers. To test this hypothesis, we measured the rate of proton extrusion in epimastigotes and found a significantly higher rate of proton extrusion in dKO parasites (Figure 6C red line) compared with WT (Figure 6C black line). Rate of extrusion measured in extracellular buffer lacking K^+^ was reduced by 34% both in WT and dKO parasites (Figure 6D white bars) and was almost completely eliminated in absence of Na^+^ (Figure 6D gray bars) or when both ions were replaced by NMDG (Figure 6D striped bars). This provides strong evidence of the presence of active ion exchangers in *T. cruzi*.

#### Intracellular Calcium Levels

**Figure 7:**
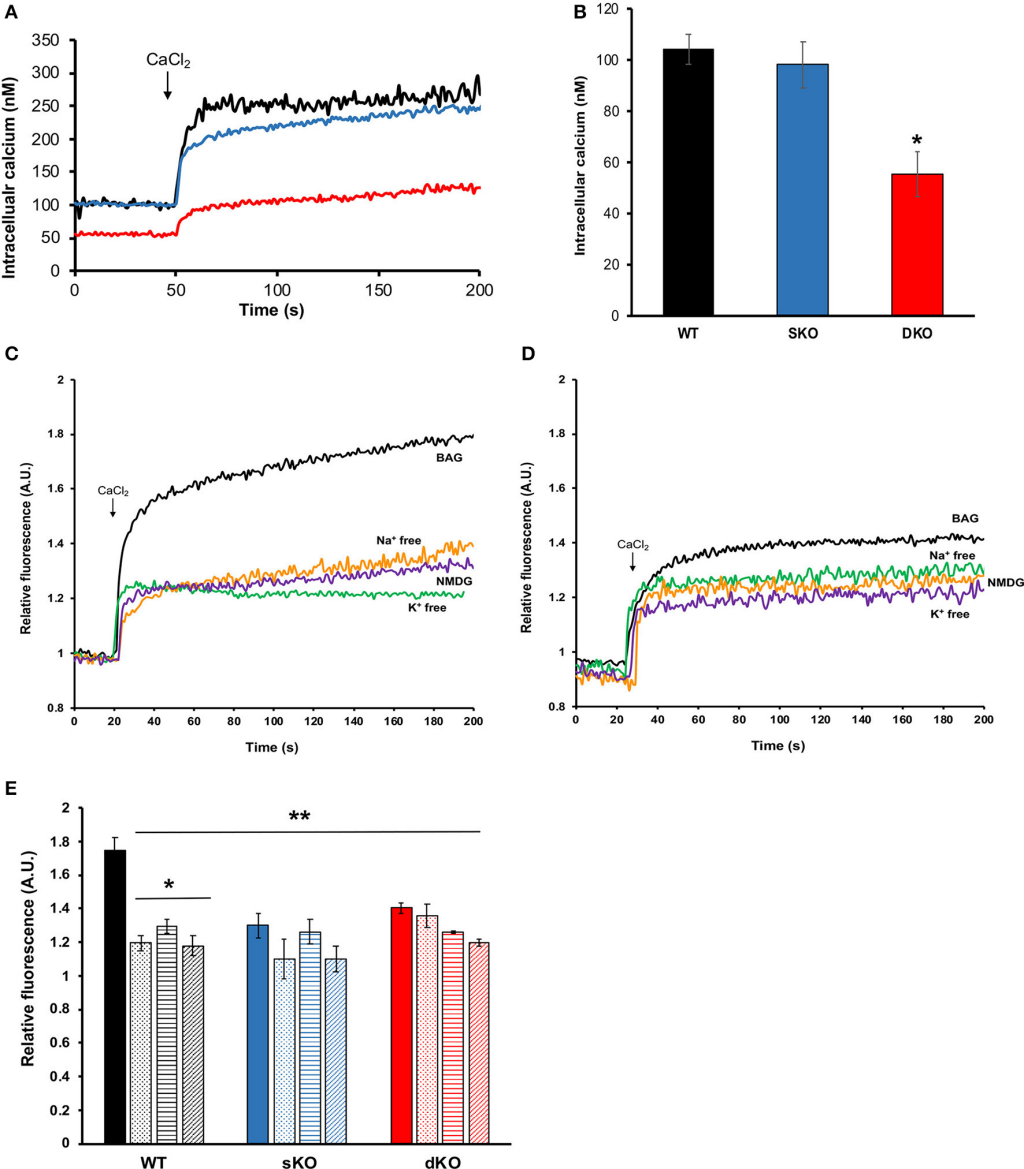
Intracellular calcium measurements. **(A)** Representative traces of Fura-2 AM loaded epimastigotes showing intracellular calcium concentrations for WT (black trace), sKO (blue trace) or dKO (red trace) in BAG. At 50 s of recording, 1.8 mM CaCl_2_ was added to the extracellular buffer. **(B)** Quantification of baseline intracellular calcium in BAG prior to extracellular calcium addition. Values are Mean ± SD of 5 independent experiments. ^*^p < 0.05 compared with WT. **(C,D)** Representative traces of intracellular calcium measurements (in relative fluorescence units for comparison purposes) of WT **(C)** and TcCAKS dKO **(D)**, in the indicated buffer conditions. **(E)** Intracellular calcium changes (in relative units) upon CaCl_2_ addition in WT (black), sKO (blue), and dKO (red) epimastigotes in BAG (solid bars), Na^+^ free (dotted bars), K^+^ free (horizontal stripe bars) or NMDG buffer (striped bars). Values are Mean ± SD of 5 independent experiments. ^*^p < 0.05 compared with BAG, ^**^p < of each condition and strain compared with WT in BAG. Statistical analysis for multiple conditions was done by two-way ANOVA with *post-hoc* Bonferroni correction.

Calcium plays a central role in *T. cruzi*, regulating cell infectivity (Moreno et al., 1994; Caradonna and Burleigh, 2011) and signaling (Burleigh and Woolsey, 2002; Docampo and Huang, 2014). Intracellular calcium balance depends on strict membrane potential regulation as the main plasma membrane permeation pathways are voltage-gated calcium channel (Verheugen et al., 1995; Christel and Lee, 2012; Harraz and Altier, 2014). Thus, we investigated whether TcCAKC function affects calcium homeostasis in the parasites. Fluo2-AM loaded dKO epimastigotes in BAG had a lower steady state intracellular calcium (55.4 ± 8.84 nM) compared with WT (105 ± 6.69 nM) and sKO (98.5 ± 9.05 nM) as it is shown in Figures 7A,B. Upon addition of 1.8 mM extracellular calcium, WT and sKO show a robust increase in cytosolic calcium (Figure 7A black and blue lines, respectively), while dKO have a modest increase (Figure 7A red line), reaching levels similar to WT under baseline conditions (first 50 s). Given the significant difference in intracellular calcium observed in the TcCAKC dKOs, we compared calcium concentrations under ionic replacement conditions by normalizing the values respect to the initial fluorescence ratio for each cell line. WT epimastigotes show a reduced cytosolic calcium increase when under Na^+^ free, K^+^ free or NMDG conditions (Figure 7C and Table 3) indicating that, at least 60% of the increase is dependent of monovalent cations. This effect can be attributed to direct activity of channel and exchangers or indirectly through decrease of the open probability of voltage-gated calcium channels, as we have observed that in absence of monovalent cations epimastigotes are hyperpolarized (Figure 5B). As expected, the cytosolic calcium levels stayed lower in dKOs under all ionic conditions (Figures 7D,E and Table 3).

**Table 3:** Baseline [Ca^2+^]_intra_ in epimastigotes.

	**WT**	**sKO**	**dKO**
BAG	105 ± 6.69	98.5 ± 9.05	55.4 ± 8.84^*^
Na^+^ Free	35.2 ± 19.7^#^	54.3 ± 4.92^#^	44.9 ± 10.1
K^+^ Free	35.8 ± 7.39^#^	55.1 ± 5.35^#^	71.7 ± 11.34
NMDG	14.7 ± 2.67^#^	58.5 ± 11.4^#^	25.4 ± 9.13

*Intracellular Ca^2+^ concentrations ([Ca^2+^]-_intra_ in nM) of epimastigotes in various ionic environments. Fura-2AM loaded epimastigotes were used to measure [Ca^2+^]_intra_ Baseline data is average [Ca^2+^]_intra_ for first 50 s of recording. Data presented as M±SD*.

* *p < 0.05 when compared to the WT strain in the same condition*.

*,# *p < 0.05 when compared to standard conditions in the same strain. Multiple conditions were analyzed by ANOVA with post-hoc Bonferroni correction. N = 5 for all experiments*.

In summary, ablation of TcCAKC has a profound effect on cellular homeostasis, with epimastigotes showing a significant hyperpolarization, increase in intracellular pH and rate of proton extrusion, and decrease in cytosolic Ca^2+^concentrations. These results, together with the observed reduction in growth rate and the inability to produce sustained infection in mammalian cells supports the role of TcCAKC as key regulator of *T. cruzi* physiological fitness.

## Discussion

Ion channels properties and their roles in a diverse range of cellular functions have been extensively studied in mammalian cells and bacteria. Surprisingly, much less information is available regarding ion channel function in other organisms and especially in protozoan parasites. Ion channels show highly conserved functional domains but the overall sequence identity is low, making difficult their finding by bioinformatics methods. At the same time, their divergence from channels present in mammalian hosts provide a unique opportunity for the development of selective new drugs.

This work provides molecular and functional evidences of the expression of a calcium-activated potassium channel required for parasite growth and infectivity. TcCAKC shares general structural features with other CAKCs: a 6 transmembrane domain topology, with a highly conserved selectivity filter (TVGYG) in the loop between TM5 and TM6 and a long C terminal domain. Unlike CAKCs of large conductance (BK), TcCAKC does not have Ca^2+^ binding sites known as “calcium bowls” Instead, putative calmodulin binding domains were predicted at the C- and N terminal ends of the protein. This is characteristic of CAKCs of intermediate and low conductance, which activation depends on calcium-calmodulin binding (Kaczmarek et al., 2017; Sforna et al., 2018). TcCAKC was expressed in all three main life stages of the parasites and colocalized with calcium-binding proteins FCaBP and calmodulin, providing further support of its activation via calcium-calmodulin complexes.

TbK1, the *T. brucei* homolog previously described does not possess a conserved selectivity filter and requires dimerization with TbK2 to produce significant currents when expressed in *X. laevis* oocytes (Steinmann et al., 2015). In contrast, TcCAKC expression and analysis by two-electrode voltage clamp produced robust and reproducible currents that require activation by calcium, indicating that *TcCAKC* encodes an α-pore forming subunit able to traffic and assemble into functional channels. No accessory β-subunits of K^+^ channels have been identified in *T. cruzi*, but recent reports have shown that KHARON, a protein complex unique to trypanosomatids, is required for correct targeting of calcium in *T. brucei* (Sanchez et al., 2016) and glucose transporters in Leishmania (Tran et al., 2013), highlighting a non-canonical pathway for membrane protein trafficking in these parasites.

The electrophysiological characterization of TcCAKC confirms its calcium-dependency and our results show that either intra or extracellular pools can be used as a source for activation of the channel. Interestingly, barium significantly decreases the current elicited by TcCAKC, while other typical K^+^ channel blockers such as 4-AP and TEA had no significant effect. Barium has been described as a potent modulator with activation and blockage effects depending on cell and channel types (Inomoto and Tokimasa, 1998; Zhou et al., 2012; Wrighton et al., 2015; Kourghi et al., 2017), but its lack of selectivity precludes its use as a potential therapeutic drug. Recently, it has been shown that combination of antibiotics with zinc restore the susceptibility of antibiotic resistant Gram (-) bacteria (Magallon et al., 2019). This approach is worth of consideration, as the combination of anti-parasitic drugs of low efficacy with barium or other less toxic metals could increase the susceptibility of the parasites and provide new avenues for drug development.

In the parasites, TcCAKC knockout has a dramatic effect on the growth of epimastigotes and impairs infectivity, with a reduction of the number of intracellular amastigotes and lack of production of tissue-derived trypomastigotes. It is well-documented the role that K^+^ channels play in regulating cell proliferation in tumoral (Liu et al., 2017; Steudel et al., 2017) and non-tumoral cells (He et al., 2011; Urrego et al., 2014). CAKCs control the progression of the cell cycle by mechanisms associated with K^+^ permeation and by signaling pathways activated independently of the channel pore activity (reviewed in Urrego et al., 2014). Coordinated oscillation of K^+^ channels expression and activity are linked to the expression of cyclins. Moreover, hyperpolarization of cells causes arrest in G1/S checkpoint and decreases cell proliferation (Wonderlin et al., 1995; Márián et al., 2000; Ouadid-Ahidouch et al., 2004). Activation of K^+^ potassium channels also maintains an electrochemical gradient that favors Ca^2+^ influx into the cells, regulating proliferation through signaling (Lee et al., 1993; Lin et al., 1993; Lallet-Daher et al., 2009). As we evaluated the phenotype of TcCAKC dKO cells, we found a significant decrease in the resting membrane potential toward hyperpolarized potentials and a reduction in cytosolic Ca^2+^ concentration. It is plausible to think that TcCAKC is playing similar roles in *T. cruzi* as the ones described in other cell types where CAKCs regulate cell replication rates. Our results demonstrate that, while the channel participates in resting membrane potential maintenance, K^+^ is not the primary driver of the electrical gradient across membranes as cells are able to maintain their membrane potential in absence of this ion. This agrees with previous evidences showing the role of H^+^ and Na^+^ ATPases in *T. cruzi* membrane potential regulation (Van Der Heyden and Docampo, 2002). Van der Heyden et al. also demonstrated that increase in extracellular K^+^ causes depolarization (Van Der Heyden and Docampo, 2002). Since the theoretical V_eq_ for K^+^ in the recording conditions was −85 mV, K^+^ would flow inward to the cell through conductive pathways, so the loss of TcCAKC would cause hyperpolarization due to less positive charge build-up in the cytosol. Membrane potential and intracellular pH homeostasis are linked with one another in *T. cruzi* due to the regulation of membrane potential by H^+^ ATPases located in the membrane (Van Der Heyden and Docampo, 2000). TcCAKC deletion caused significant alkalization compared to that of WT or sKO parasites, which correlates with the hyperpolarization of dKO cells. A similar link between these two parameters has been shown in *Arabidopsis* (Gambale and Uozumi, 2006). Ba^2+^ treatment only caused acidification in WT but not in sKO or dKOs parasites, also suggesting that pH regulation is partially K^+^ dependent. Membrane potential and changes in intracellular pH were accompanied by an increase in the rate of proton extrusion in the dKO compared with WT parasites. The rate of proton extrusion was moderately decreased in absence of K^+^, but practically abolished in absence of Na^+^ arguing about the presence of Na^+^/H^+^ and K^+^/H^+^ exchangers. Biochemical evidences support the presence of these transporters in *T. cruzi* (Van Der Heyden and Docampo, 2002; Gil et al., 2003), and at least 1 gene encoding for a putative Na^+^/H^+^ antiporter (TcCLB.510511.9) is present in the *T. cruzi* genome, but no expression or functional evidences have been reported.

**Figure 8:**
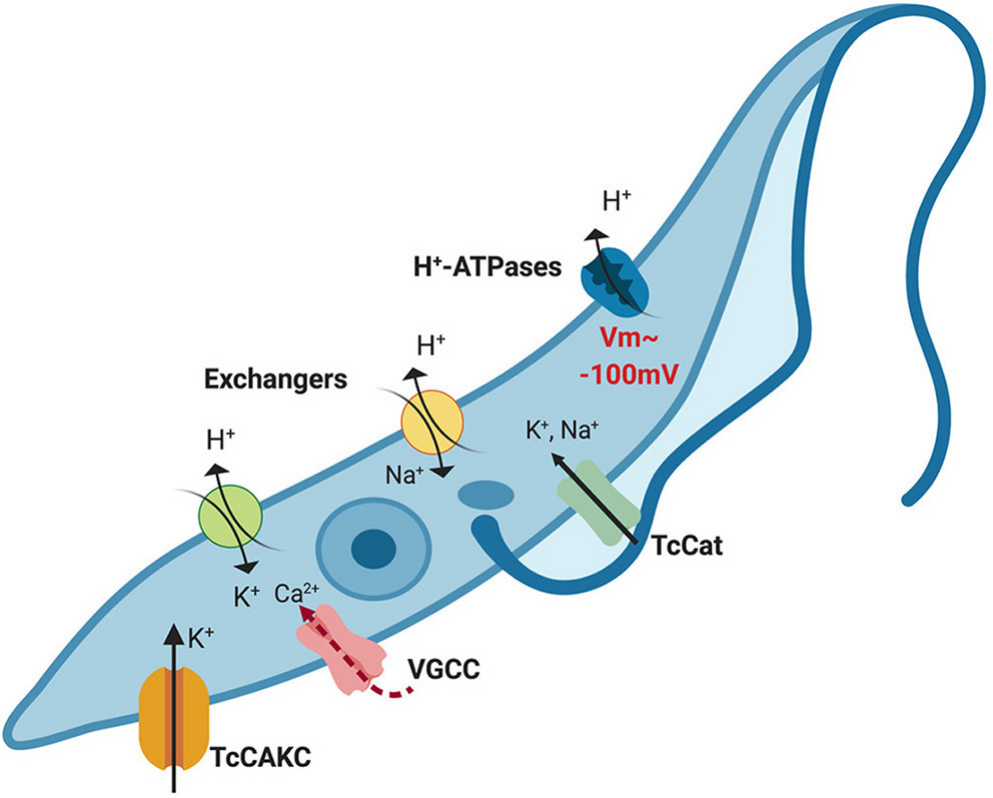
Proposed model of ionic homeostasis in *T. cruzi*. H^+^ ATPases maintain the gradient of protons that drive the resting membrane potential, which is about −100 mV in epimastigotes. Additionally, Na^+^/H^+^ and K^+^/H^+^ exchangers contribute to H+ extrusion mobilizing other monovalent ions. Intracellular potassium concentration is balanced through the combined action of channels and exchangers. Given the negative membrane potential beyond the theoretical equilibrium potential for K^+^ (~80 mV), activation of TcCAKC can mediate K^+^ efflux until the membrane potential reaches values higher than the equilibrium potential, when the electrochemical gradient will drive K^+^ out of the cells. Depolarization of the membrane could then activate voltage-gated calcium channels (VGCC), responsible for oscillations of the intracellular Ca^2+^ concentration. For simplicity purposes, in this model we have omitted PMCA and SERCA-like pumps that participate in intracellular calcium homeostasis.

The evidences found in the literature together with the results reported here are drawing a clearer picture of how *T. cruzi* regulates ionic homeostasis (Figure 8). The resting membrane potential is maintained primarily by H^+^ ATPases and only partially supported by K^+^ (Van Der Heyden and Docampo, 2002). The activity of these ATPases is possible due to proton gradients maintained by metabolic activity and exchangers such as Na^+^/H^+^ and K^+/^H^+^ antiporters. Yet, the intracellular environment is abundant in K^+^ that can be mobilized via non-selective cation channels (Jimenez and Docampo, 2012), TcCAKC and most probably other K^+^ channels (Jimenez et al., 2011). With a resting membrane potential close to −100 mV, TcCAKC could be mediating K^+^ influx that buffers the hyperpolarization effect caused by H^+^ efflux and activates voltage gated Ca^2+^ channels. In the absence of TcCAKC the reduction in inward currents will cause a shift in membrane potential to more negative values, alkalinization of the cytosol and decrease in calcium levels. As consequence of inadequate ionic homeostasis and changes in membrane potential, the replication rate and infectivity of the parasites is severely impaired. This working model provides us with a roadmap to keep interrogating canonical and non-canonical functions of ion channels in trypanosomatids and to explore new avenues for drug development targeting these unique proteins.

## Data Availability Statement

The datasets generated for this study are available on request to the corresponding author.

## Author Contributions

PB and ND were responsible for the design and execution of the mutant and knockout cell lines. CS and MB performed growth assays, electrophysiological recordings, and fluorometric measurements. VJ designed and directed the study, analyzed data, provided funding, and wrote the manuscript.

### Conflict of Interest

The authors declare that the research was conducted in the absence of any commercial or financial relationships that could be construed as a potential conflict of interest.
