# Corrigendum: A Novel Calcium-Activated Potassium Channel Controls Membrane Potential and Intracellular pH in *Trypanosoma cruzi*

**DOI:** 10.3389/fcimb.2021.742256

**Published:** 2021-10-19

**Authors:** Patricia Barrera, Christopher Skorka, Michael Boktor, Noopur Dave, Veronica Jimenez

**Affiliations:** ^1^ Departmento de Biología, Facultad de Ciencias Exactas y Naturales, Instituto de Histologia y Embriologia IHEM-CONICET, Facultad de Medicina, Universidad Nacional de Cuyo, Mendoza, Argentina; ^2^ Department of Biological Science, College of Natural Sciences and Mathematics, California State University Fullerton, Fullerton, CA, United States

**Keywords:** potassium channel, intracellular calcium, membrane potential, electrophysiology, *Trypanosoma cruzi*

## Incorrect Affiliation

In the published article, there was an error in affiliation 1 and 2. Instead of “Patricia Barrera^1^”, it should be “Patricia Barrera^2^” and instead of “Christopher Skorka^2^, Michael Boktor^2^, Noopur Dave^2^ and Veronica Jimenez^2^” it should be “Christopher Skorka^1^, Michael Boktor^1^, Noopur Dave^1^ and Veronica Jimenez^1^.

The authors apologize for this error and state that this does not change the scientific conclusions of the article in any way. The original article has been updated.

## Publisher’s Note

All claims expressed in this article are solely those of the authors and do not necessarily represent those of their affiliated organizations, or those of the publisher, the editors and the reviewers. Any product that may be evaluated in this article, or claim that may be made by its manufacturer, is not guaranteed or endorsed by the publisher.

