# Supplementary figures and images for: A Novel Calcium-Activated Potassium Channel Controls Membrane Potential and Intracellular pH in Trypanosoma cruzi
Source: Front Cell Infect Microbiol. 2020 Jan 15;9:464. doi: 10.3389/fcimb.2019.00464 (PMC6974456; doi:10.3389/fcimb.2019.00464)

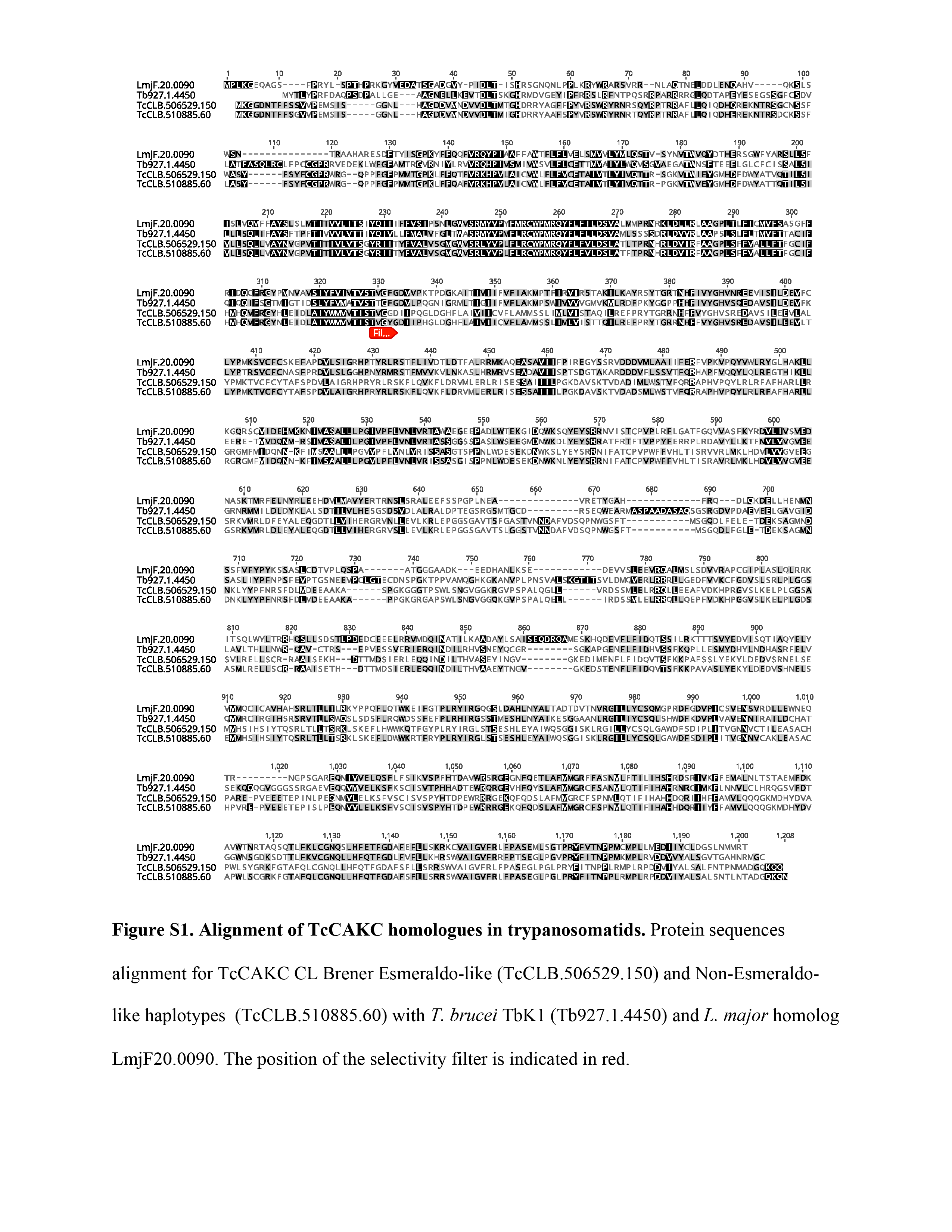

Supplement: Supplementary file 1 [file Image_1.TIF]

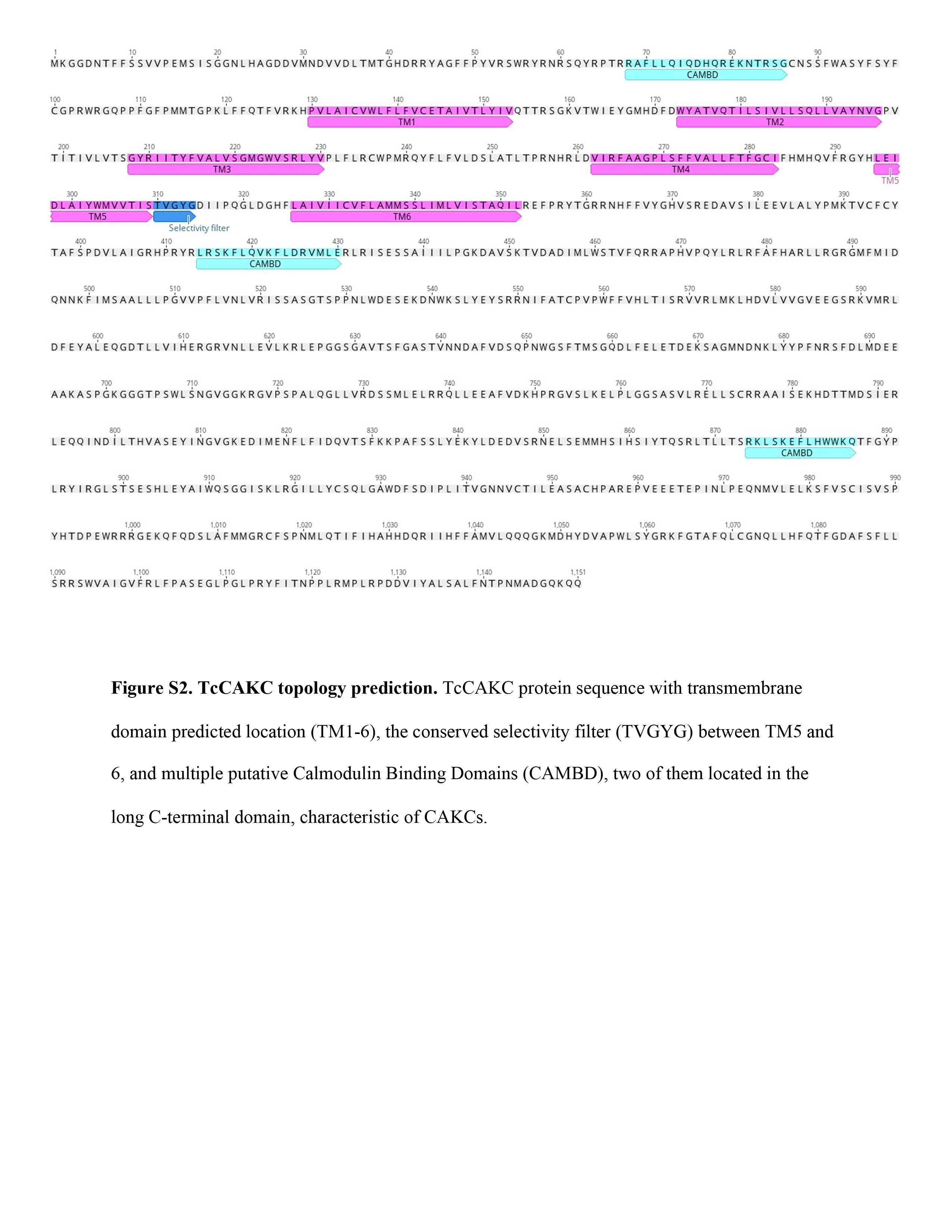

Supplement: Supplementary file 2 [file Image_2.TIF]

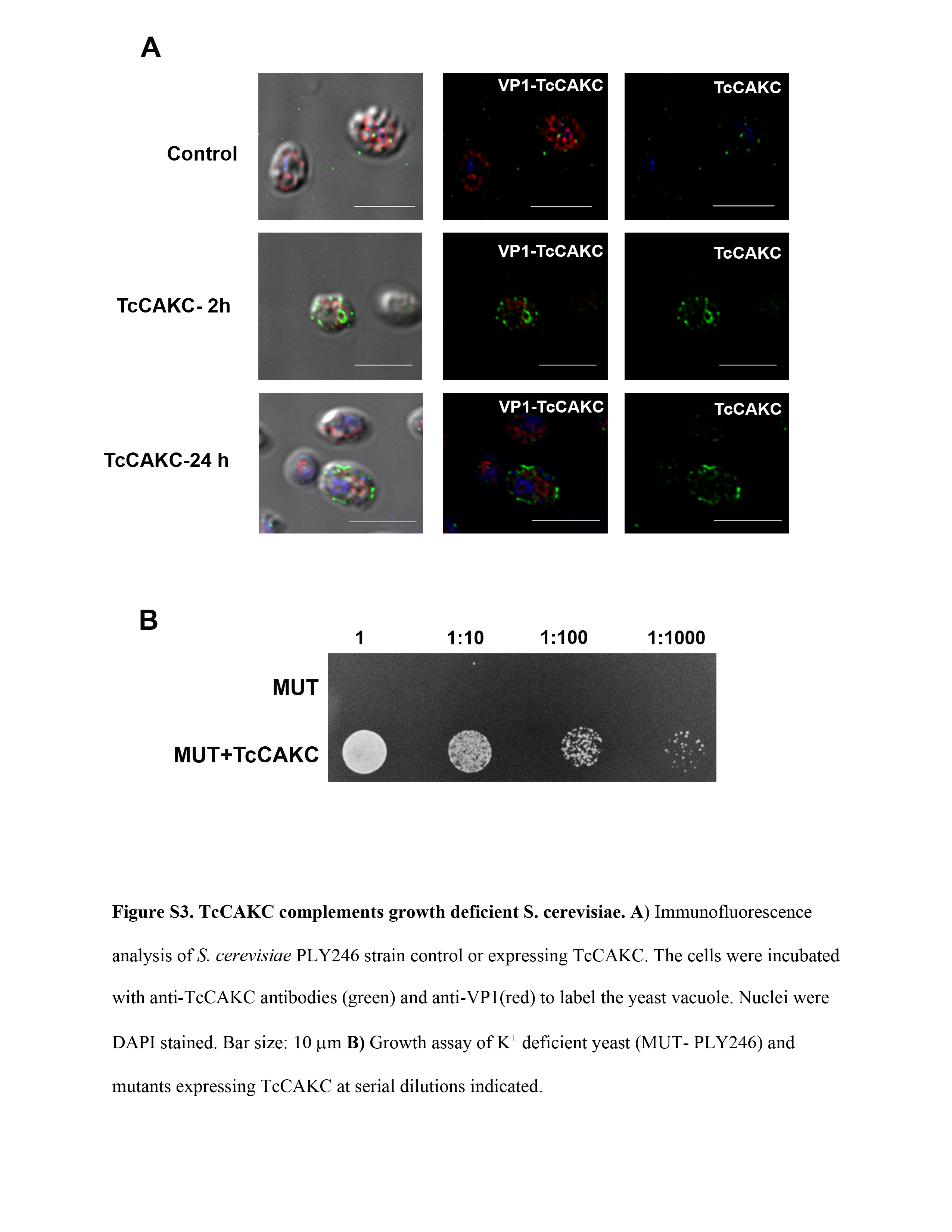

Supplement: Supplementary file 3 [file Image_3.TIF]
